# Identifying the key microbiome associated with lumbar discherniation in Chinese patients

**DOI:** 10.3389/fcell.2026.1942642

**Published:** 2026-09-15

**Authors:** Xiaoguang Han, Zhou Dan, Qinling Mo, Xin Rao, Yong Chen, Yunhe Jiang, Changwei Li, Guoqing Tang

**Affiliations:** 1 Department of Spine Surgery, Beijing Jishuitan Hospital, Beijing, China; 2 Shanghai Key Laboratory for Prevention and Treatment of Bone and Joint Diseases, Department of Orthopedics, Shanghai Institute of Traumatology and Orthopedics, Ruijin Hospital, Shanghai Jiao Tong University School of Medicine, Shanghai, China; 3 Department of Central Laboratory, Kunshan Hospital of Chinese Medicine, Affiliated Hospital of Yangzhou University, Suzhou, Jiangsu, China; 4 Department of Oncology, Kunshan Hospital of Chinese Medicine, Affiliated Hospital of Yangzhou University, Suzhou, Jiangsu, China; 5 School of Pharmacy, Nanjing University of Chinese Medicine, Nanjing, Jiangsu, China; 6 Department of Orthopedics, Kunshan Hospital of Chinese Medicine, Affiliated Hospital of Yangzhou University, Suzhou, Jiangsu, China; 7 Department of Clinical Laboratory, Kunshan Hospital of Chinese Medicine, Affiliated Hospital of Yangzhou University, Suzhou, Jiangsu, China

**Keywords:** *Bacteroides*, diagnostic biomarker, *faecalibacterium*, gut microbiota, lumbar dis cherniation, metabolites

## Abstract

**Background:**

Inflammation and subsequent fibrotic remodeling—characterized by extracellular matrix deposition and myofibroblast activation—are hallmark processes in various degenerative disorders. Lumbar disc herniation (LDH) involves local inflammation and disruption of extracellular matrix organization; however, the role of systemic modulators such as gut microbiota and their metabolites remains poorly understood, particularly in the Han Chinese population.

**Methods:**

A total of 69 LDH patients and 69 healthy controls were enrolled in this study. Fecal samples were subjected to 16 S rRNA sequencing, and untargeted metabolomics was performed to compare microbial diversity, taxonomic composition, and metabolic profiles between the two groups. A random forest model was constructed to evaluate the diagnostic predictive value of identified microbial and metabolic features.

**Results:**

LDH patients exhibited significant gut microbial dysbiosis, characterized by reduced alpha and beta diversity and markedly decreased abundances of Faecalibacterium and *Bacteroides*. These microbial alterations were associated with chronic inflammation driven by elevated proinflammatory factors, suppression of glutamatergic and GABAergic neuronal signaling, and dysregulation of pathways involved in mannan degradation and cytoskeleton assembly—processes closely linked to cell–matrix interactions and fibrotic tissue remodeling. Metabolites including phenylalanine and beta-alanine were identified as potential regulatory molecules. The random forest model incorporating microbial pathways and metabolites demonstrated good diagnostic accuracy for distinguishing LDH patients from healthy controls.

**Conclusion:**

These findings suggest that gut microbiota may contribute to intervertebral disc degeneration by promoting chronic inflammatory responses and fibrotic structural remodeling. This study provides new insights into the inflammation–fibrosis continuum underlying spinal degeneration and identifies candidate biomarkers with potential for future translational applications.

## Introduction

Lumbar disc herniation (LDH) is a common disease worldwide and often occurs at ages 30 and 50. LDH has a prevalence of 5% in adults, and the incidence rate in males is twofold greater than that in females (Penchev et al., 2024; KailaKangas et al., 2009). For this disease, the lumbar disc nucleus pulposus outflows from the intervertebral space, which causes back pain (van der Windt et al., 2010). Lumbar disc degeneration (LDD) is a key contributor to LDH (Shenoy et al., 2019). As patients age, the nucleus pulposus undergoes senescence and degenerative processes, which ultimately lead to nucleus pulposus outflow, causing pain due to nerve compression (Shi et al., 2019). The main treatment strategies for LDH include conservative and surgical interventions (Nakajima et al., 2024).

Conservative treatments are usually used for patients with mild symptoms. Nonsteroidal anti-inflammatory drugs (NSAIDs) or opioid analgesics are the most common drugs used for LDH treatment (Xia et al., 2024; Zhou et al., 2023), and physical therapy can also alleviate initial LDH symptoms (Wang et al., 2022). Surgery is the only method for advanced-stage LDH treatment, and some reports have shown that surgery may be more effective than conservative treatment (Lurie et al., 2014). However, a 13-year follow-up study has revealed that 8% of patients who had undergone surgery for LDH required revision surgery, whereas 14% of those treated conservatively underwent spinal surgery (Nykvist et al., 1995). Therefore, a deeper investigation into the mechanism and drug targets for LDH treatment is necessary.

The gut microbiota is an excellent candidate for explaining the mechanisms underlying LDH, as it plays a crucial role in various physiological and pathological conditions. Some reports have shown that depletion of the gut microbiota by antibiotics abolishes mechanically induced increases in bone mass (Wang et al., 2024). The gut microbiota of lean mice has the potential to decrease body weight (Ridaura et al., 2013; Ng et al., 2022), and transplantation of gut microbiota from young mice reverses hallmarks of aging in old mice (Parker et al., 2022). These results suggest that alterations in the gut microbiota and metabolites may influence the progression of various diseases, including LDH.

*Faecalibacterium* and *Bacteroides* are well-known probiotics in the gut. Numerous studies have reported that low levels of *Faecalibacterium* and *Bacteroides* are correlated with inflammatory conditions, including inflammatory bowel disease (Cabezas-Cruz and Bermúdez-Humarán, 2024; Brown et al., 2019) and rheumatoid arthritis (Moon et al., 2023). Given that LDH is associated with tissue senescence and inflammatory infiltration (Yu et al., 2022; Ozden and Silav, 2023), the alteration of *Faecalibacterium* and *Bacteroides* in the gut may play a key role in LDH progression. Obesity is among the most important factors that induce LDH, and decreases in *Faecalibacterium* and *Bacteroides* have also shown promise in preclinical models of obesity and obesity-associated disorders (Vallianou et al., 2023), suggesting that *Faecalibacterium* and *Bacteroides* may affect LDH by increasing body weight. In addition, low levels of *Faecalibacterium* also lead to a decrease in SCFA production, promoting anxiety and depression-like behaviors in alcohol-dependent patients (Li C. et al., 2024). Some studies have reported that anxiety is an independent predictor of older patients undergoing surgery for lumbar disc herniation (Mou et al., 2023). These results suggest that alterations in the abundances of *Faecalibacterium* and *Bacteroides* are key factors in LDH initiation and progression.

Although short-chain fatty acids (SCFAs) are the major metabolites of *Faecalibacterium* and *Bacteroides* involved in the progression of various diseases (Strasser and Ticinesi, 2023; Rees et al., 2022), the present study identified new metabolites derived from *Faecalibacterium* and *Bacteroides*, such as phenylalanine and β-alanine, which have never been reported to be involved in LDH progression. These metabolites play important roles in neurodegenerative diseases (Murakami et al., 2023) and inflammation-related diseases (Eicher and Mohajeri, 2022), which are related to LDH. Thus, these metabolites may provide new insights into the mechanisms of LDH, thereby aiding the search for novel drug targets for LDH treatment.

## Materials and methods

### Ethics approval and statement

This study was approved by the Ethics Committee of Kunshan Hospital of Chinese Medicine, Affiliated Hospital of Yangzhou University Affiliated Hospital of Yangzhou University (Ethics number: KZY 2023-005-02). All the participants agreed and signed an informed consent for fecal sample donation before collection.

### Sample collection and cohort description

To explore the characteristics of the gut microbiome in LDH, 69 patients (average age of 56.4 ± 1.79; 32 males and 37 females) who were diagnosed with LDH were recruited. In addition, 69 healthy individuals (average age of 55.4 ± 1.80; 35 males and 34 females) were included in the control cohort.

### Study cohort recruitment and fecal sample collection

The participants with LDH in this study were diagnosed according to the current diagnostic and treatment strategies for intervertebral disc disease (Wu et al., 2020). The participants were recruited from Suzhou, Jiangsu Province, China. The exclusion criteria were as follows: abnormal cognition (independent ability to complete the questionnaire about the Mini-Mental State Examination); other severe intervertebral disc-related illnesses or other severe bone-related diseases (rheumatoid arthritis or osteoarthritis diseases); serious chronic diseases (hypertension, fat or neurodegenerative disorders), acute or chronic gut diseases (diarrhea or Crohn’s disease), and cancers or acute infectious diseases (pneumonia) (Pang et al., 2023). Moreover, all the subjects in the present study had not taken antibiotics, probiotics or prebiotics within 3 months before stool collection. According to the collection and storing requirements, the fecal samples were collected at the hospital, divided into two parts (200 mg each) and immediately stored at −80 °C.

### DNA extraction, amplification and sequencing

Total bacterial DNA was isolated using a Fast DNA Spin Kit for Feces (MP Biomedicals, USA) according to the manufacturer’s instructions. The concentration and purity of the extracted DNA were assessed using a NanoDrop NC2000 spectrophotometer (Thermo Fisher Scientific, USA) and agarose gel electrophoresis, respectively. Universal primers 338F (5′-ACT​CCT​ACG​GGA​GGC​AGC​A-3′) and 806R (5′-GGACTACHVGGGTWTCTAAT-3′) were used to amplify the V3–V4 hypervariable region of the bacterial 16S rRNA gene. Sample-specific 7-bp barcodes were incorporated into the primers for multiplex sequencing. PCR amplification was performed in a 25 µL reaction mixture containing 5 µL of 5× buffer, 0.25 µL of FastPfu DNA Polymerase (5 U/μL), 2 µL of dNTPs (2.5 mM each), 1 µL of each forward and reverse primer (10 µM), and approximately 25 ng of fecal DNA, with sterile water added to a final volume of 25 µL. The thermal cycling protocol consisted of an initial denaturation at 98 °C for 5 min, followed by 25 cycles of 98 °C for 30 s, 53 °C for 30 s, and 72 °C for 45 s, with a final extension at 72 °C for 5 min, and then held at 4 °C. PCR products were purified using VAHTS DNA Clean Beads (Vazyme, China) and quantified with the Quant-iT PicoGreen dsDNA Assay Kit (Invitrogen, USA). Equimolar amounts of purified amplicons were pooled, and paired-end sequencing (2 × 250 bp) was performed on an Illumina NovaSeq 6000 platform using the NovaSeq 6000 SP Reagent Kit (500 cycles) at Shanghai Personal Biotechnology Co., Ltd. (Shanghai, China).

### 16S rRNA data processing

Raw sequencing data in FASTQ format were processed using the QIIME 2 (2022.11) platform (Bolyen et al., 2019), which integrates a suite of plugins including demux, cutadapt, and DADA2. Prior to analysis, sequence reads were demultiplexed using the demux plugin, and primer sequences were removed using the cutadapt plugin (Kechin et al., 2017). Denoising, quality filtering, read merging, and chimera removal were subsequently performed using the DADA2 plugin (Callahan et al., 2016). Amplicon sequence variants (ASVs) were inferred at 100% sequence identity, and only non-singleton ASVs were retained for downstream abundance analyses.

### Rarefaction curve, diversity and taxonomy analysis

Rarefaction analysis was performed using the vegan package (version 2.6–4) in R to evaluate species richness based on sequencing data from the NC and LDH cohorts. Alpha diversity indices—including ACE, Chao1, Richness, Pielou’s evenness, Shannon diversity index, Simpson index, and Good’s coverage—were calculated using the vegan package based on ASV abundance. Beta diversity was assessed by principal coordinate analysis (PCoA) using the phyloseq package (version 1.46.0) in R (McMurdie and Holmes, 2015), based on Bray-Curtis, weighted UniFrac, and unweighted UniFrac distance metrics. Linear discriminant analysis effect size (LEfSe) was employed to identify microbial taxa with differential abundance between the two groups, using the Wilcoxon rank-sum test (P < 0.05) and linear discriminant analysis (LDA) scores (LDA >2) as thresholds for biomarker selection (Segata et al., 2011). Visualization of differential microbial abundance was performed using heatmaps generated with the Complex Heatmap package (Gu, 2022), Sankey diagrams with the ggalluvial package (Brunson, 2020), and a circular phylogenetic tree combined with heatmaps, boxplots, and dot plots using the ggtreeExtra package (Xu et al., 2021).

### Co-occurrence network analysis

A gut microbial co-occurrence network was constructed on the basis of ASVs or genera from the NC or LDH group via the WCGNA and igraph packages in R. ASVs or genera with a relative abundance (RA) higher than 0.01% and occurring in more than 1/10 of the samples in each group were included for analysis. Spearman’s correlations of fecal microbes in the NC or LDH samples were conducted on the basis of the relative abundance of ASVs and genera to construct a co-occurrence network. The selected ASVs (Spearman’s correlation coefficient (Rho) > 0.5 and FDR <0.05) or genera (Rho >0.5 and FDR <0.05) were visualized by Gephi software (version 0.10). To evaluate the similarity of the co-occurrence network, topological features, such as the weighted degree, betweenness centrality, clustering and statistical inference, were calculated by Gephi, and differences were tested via the Wilcoxon rank-sum test and plotted via the ggplot2 package in R. To understand the topological roles of the ASVs (nodes) in the co-occurrence network, both within-module connectivity (Zi), which indicates that a node links to other nodes within its own module, and among-module connectivity (Pi), which reflects the linking node, were adopted and calculated following a previously reported method (Guimerà and Nunes Amaral, 2005). According to Zi and Pi, the topological role of each node was classified into the following four types: connectors (nodes with Pi > 0.62 and Zi < 2.5), module hubs (nodes with Zi > 2.5 and Pi < 0.62), network hubs (nodes with Zi > 2.5 and Pi > 0.62) and peripherals (nodes with Zi ≤ 2.5 and Pi ≤ 0.62). Network hubs, module hubs, and connectors are regarded as putative keystone microbes that are thought to play vital roles in the gut microbial community structure and potential functions (Guimerà and Nunes Amaral, 2005; Sun et al., 2022).

Similarly, on the basis of the genus-level data (RA> 0.01%, FDR <0.05) and the other variable data (such as EC numbers, KO identifiers and metabolic pathway data with RA> 0.01% and FDR <0.01), Spearman’s correlation analysis was performed between the two groups, and the genus-level and variable data (Rho >0.65 and FDR <0.05) were subsequently selected to construct a correlated network and displayed via Gephi software.

### Functional annotation

To investigate metabolic pathways on the basis of 16 S sequence data and representative ASV sequences, phylogenetic investigations of communities by reconstruction of unobserved states (PICRUSt2) (Douglas et al., 2020) were performed. Metabolic pathways were predicted via the Kyoto Encyclopedia of Genes and Genomes (KEGG) Ortholog (KO) database (Kanehisa and Goto, 2000) and the MetaCyc database (Caspi et al., 2020). The statistical differences in the relative abundances of KO identifiers, EC numbers and predicted metabolic pathways were analyzed via the Wilcoxon rank-sum test. Furthermore, the KEGG pathway information was then collapsed into the following three different levels: Category (Level 1), Super Pathway (Level 2) and Pathways (Level 3).

### Machine learning model prediction

Four types of significantly different variables—including genera-level data, alpha diversity index (ADI) data, KO identifier data, and EC number data—were randomly divided into two subsets: 75% of the data was used as a training set for the random forest (RF) model (Denisko and Hoffman, 2018), while the remaining 25% was reserved as a testing set. Based on the RF results, ten repeated rounds of 10-fold cross-validation were performed to select the optimal features ranked by MeanDecreaseGini, which achieved the best performance and improved prediction accuracy (Feng et al., 2015). The testing data were independently used to evaluate the model performance. Receiver operating characteristic (ROC) curve analysis was performed using the pROC package (version 1.18.5) in R, and the area under the curve (AUC) was calculated to assess the discriminative ability of the model (Robin et al., 2011). For RF models built using a single type of variable (i.e., genera, ADIs, KO identifiers, or EC numbers), the optimized biomarkers and the corresponding ROC curves were obtained after model prediction. Subsequently, the above-mentioned optimized features—comprising 42 microbial genera, 7 ADIs, 21 KO identifiers, and 30 EC numbers—were used in various combinations for further model prediction analyses, specifically: 42 microbial genera +7 ADIs, 42 microbial genera +21 KO identifiers, and 42 microbial genera +30 EC numbers. These combined variable sets were then evaluated using the RF model.

### Statistical analysis

The differential relative abundances of phyla, genera, ASVs, KO identifiers, EC numbers and metabolic pathways between any two groups were tested via the Wilcoxon rank-sum test using the Stats package in R (Ekstrøm and Carstensen, 2024). At test was performed for the age variable, and a chi-square test was performed for the sex variable using the Stats package in R. All the P values were corrected as false discovery rates (FDRs) with the Benjamini–Hochberg method via the Stats package in R. Distances of β diversity, KO identifiers and EC numbers were analyzed via the analysis of similarity (ANOSIM) method using the vegan package in R. P values were evaluated using the ANOSIM function on the basis of 999 permutations.

## Results

### Alteration of the gut microbiome in the LDH cohort

To characterize the gut fecal microbiome, 16S rRNA sequencing was performed. The optimal read number of all sequencing samples ranged from 29,924 to 56,932. Rarefaction curves based on the abundance of randomly sampled amplicon sequence variants (ASVs) revealed that the curves of the NC and LDH groups were close to saturation, suggesting that the sequencing data could be used for analysis and investigation (Figure 1A). AVenn diagram revealed 80 (7%) unique ASVs in the NC group, 47 (4%) unique ASVs in the LDH group, and 1,037 shared ASVs in the two groups (Figure 1B). In terms of alpha diversity, the ACE index, Shannon diversity index, Simpson diversity index, Pielou evenness index, Good’s coverage index richness index and Chao1 index of the LDH cohort were significantly lower than those of the NC cohort (Figures 1C–G; Supplementary Figures S1A,B). With respect to beta diversity, principal coordinate analyses (PCoAs) based on Bray‒Curtis, unweighted UniFrac and weighted UniFrac distance metrics revealed significant differences in microbial communities between the two cohorts (Figures 1H–J). These findings indicated that the microbial diversity in the LDH cohort significantly differed from that in the healthy group.

**FIGURE 1:**
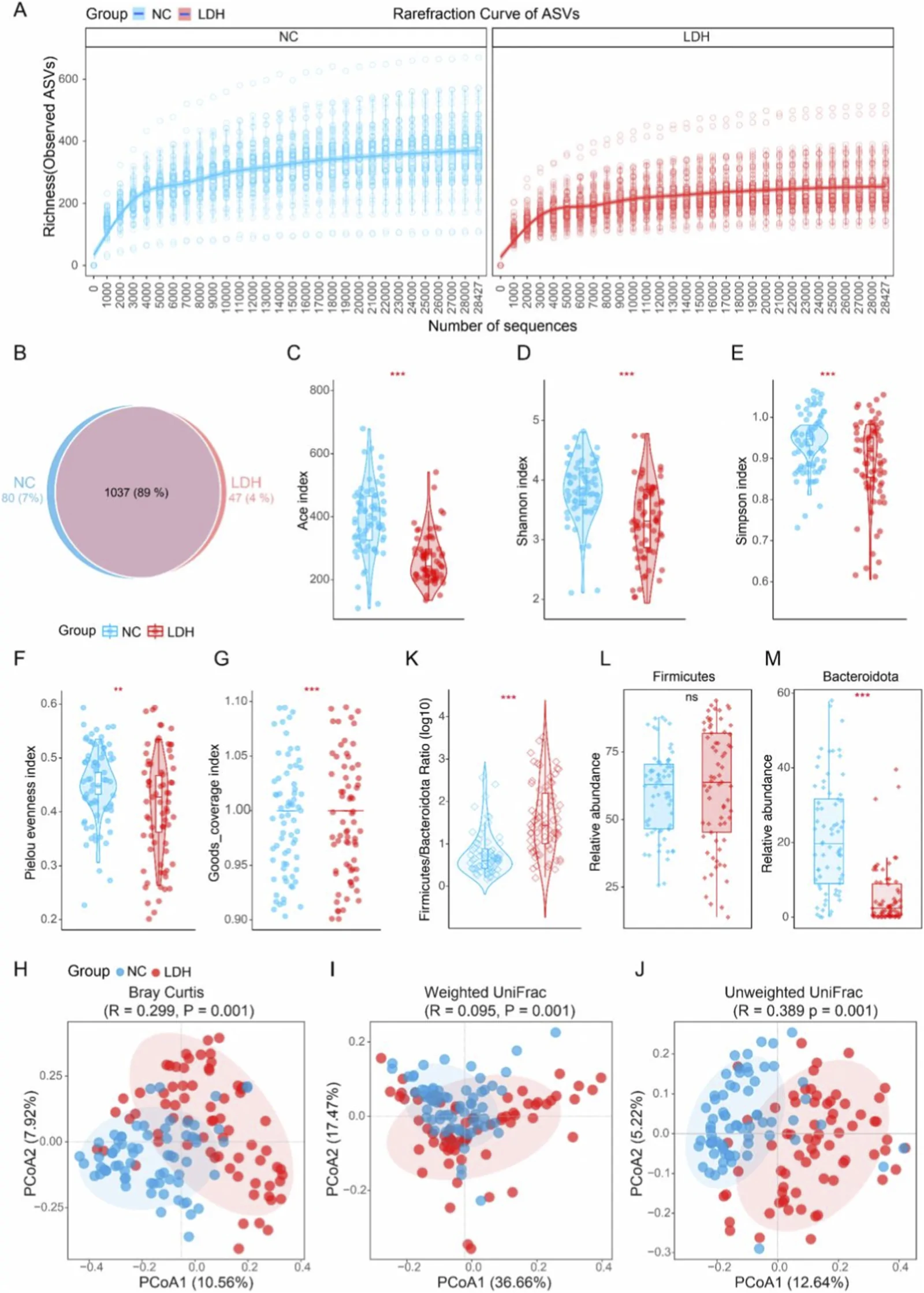
Alterations in the gut fecal microbiome between the LDH and NC cohorts. **(A)** Rarefaction curves of species richness based on amplicon sequence variants (ASVs), illustrating the sequencing depth of the 16S rRNA data. **(B)** Venn diagram showing unique and shared ASVs between LDH patients and healthy controls. **(C–G)** Alpha diversity indices, including ACE **(C)**, Shannon **(D)**, Simpson **(E)**, Pielou’s evenness **(F)**, and Good’s coverage **(G)**, comparing the two groups. **(H–J)** Principal coordinate analysis (PCoA) plots based on Bray–Curtis **(H)**, weighted UniFrac **(I)**, and unweighted UniFrac **(J)** distance metrics, with statistical significance assessed by ANOSIM. **(K)** The Firmicutes/Bacteroidetes ratio was significantly higher in the LDH group than in the NC group. **(L)** No significant difference was observed in the relative abundance of the phylum Firmicutes between the two groups. **(M)** The relative abundance of the phylum Bacteroidetes was significantly lower in the LDH cohort compared with the NC cohort. Statistical significance was determined using the Wilcoxon rank-sum test with FDR correction: *FDR <0.05, **FDR <0.01, ***FDR <0.001.

Firmicutes and Bacteroidetes are the most abundant phyla in the gastrointestinal tract, and the Firmicutes/Bacteroidetes (F/B) ratio is widely considered to play a critical role in maintaining gut homeostasis (Stojanov et al., 2020). Compared with the NC cohort, the average relative abundance of Bacteroidota in the LDH group was reduced to 5.48% (Figures 2A,B). Given that Bacteroidota was the second most predominant phylum in the NC cohort, whereas Proteobacteria ranked second in the LDH group (Figure 2A), we calculated both the F/B ratio and the Firmicutes/Proteobacteria (F/P) ratio to assess overall shifts in gut microbial composition. The F/B ratio was significantly elevated in the LDH group compared with the NC group (Figure 1K); however, no significant difference was observed in the F/P ratio between the two groups (Supplementary Figure S1C). The relative abundance of Bacteroidota was significantly higher in the NC cohort than in the LDH cohort (Figure 1M), while the abundances of Firmicutes and Proteobacteria showed only minor differences between the two cohorts (Figure 1L; Supplementary Figure S1D). These findings suggest that the reduction in Bacteroidota may contribute to the overall microbial shift toward dysbiosis in the LDH cohort.

**FIGURE 2:**
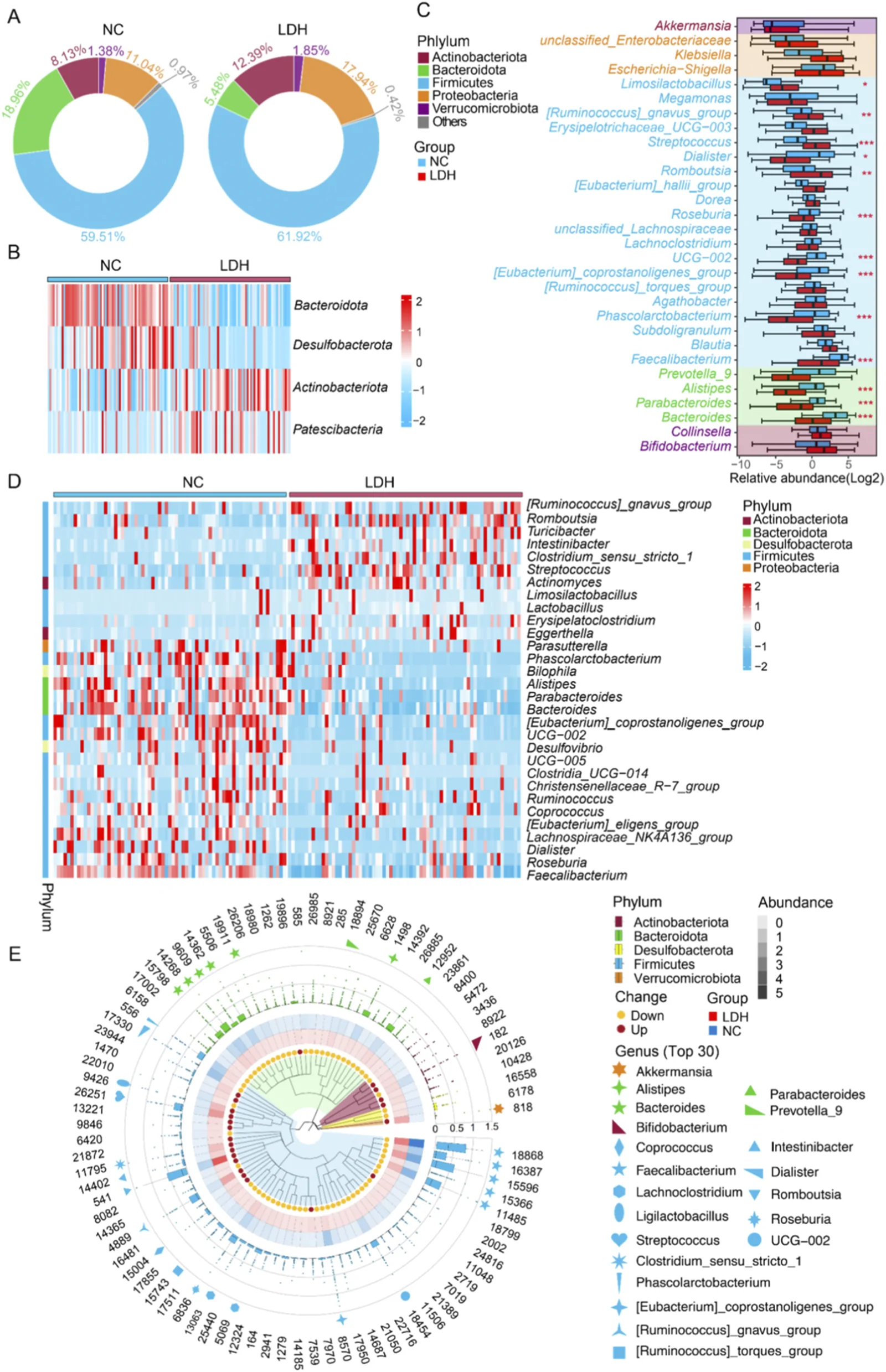
Taxonomic composition analysis of the intestinal microbiota between the LDH and NC cohorts. **(A)** Donut chart showing the relative abundance of the top 5 predominant phyla. **(B)** Heatmap of differentially abundant phyla between the two groups, with red indicating upregulation and blue indicating downregulation in the LDH group relative to the NC group; horizontal bars represent group assignments. **(C)** Boxplot of the top 30 predominant genera; red and blue boxes represent LDH and NC groups, respectively, and colors correspond to phylum-level classification. **(D)** Heatmap of the top 30 differentially abundant genera; color coding follows the same convention as in **(B)**. **(E)** Multidimensional visualization integrating a circular phylogenetic tree based on 86 differential ASVs. The layers from outside to inside represent ASV names; the top 30 ASVs are labeled with distinct shapes. The integrated display includes: boxplots showing the relative abundance of all ASVs, a heatmap depicting the average relative abundance of ASVs between groups, a dot plot representing log2-transformed fold changes (dark purple and yellow dots indicate increased and decreased abundance in the LDH group, respectively), and a circular phylogenetic tree. Colors of shapes, boxplots, and the phylogenetic tree correspond to different phyla; various shapes with distinct colors denote different genera. Statistical significance was assessed using the Wilcoxon rank-sum test with FDR correction: *FDR <0.05, **FDR <0.01, ***FDR <0.001.

### Taxonomic composition characteristics of the gut microbiota in LDH

The taxonomic composition of the gut fecal microbiota was further explored. At the phylum level, the top 5 predominant phyla, namely, *Firmicutes*, *Bacteroidota*, *Proteobacteria*, *Actinobacteriota* and *Verrucomicrobiota*, accounted formore than 99% of the overall phyla (Figure 2A). The abundance heatmap revealed 4 phyla that differed between the LDH group and the NC group (Figure 2B). The relative abundance of *Desulfobacterota* and *Bacteroidota* in the LDH group was significantly lower than that in the NC group, whereas the relative abundances of *Actinobacteriota* and *Patescibacteria* in the LDH group were significantly greater than those in the NC group (Figure 2B).

At the genus level, the top 30 predominant genera were derived primarily from the top 5 phyla, and the top 2 genera were *Faecalibacterium* and *Bacteroides* (Figure 2C). In addition, 85 genera were differentially expressed between the NC and LDH groups, including 13 genera from the top 30 predominant genera (Figure 2C). The abundance heatmap revealed the distribution of the 30 most significantly different genera across all the samples. The relative abundances of *Faecalibacterium*, *Bacteroides*, *Phascolarctobacterium* and *Roseburia* in the LDH group were significantly lower than those in the NC cohort, whereas the abundances of *Streptococcus*, *Actinomyces*, *Eggerthella*, *Ruminococcus gnavus group* and *Clostridium_sensu_stricto_1* were significantly increased in the LDH cohort compared with those in the NC cohort (Figure 2D).

At the ASV level, the top 30 predominant ASVs were derived from the top 5 phyla (21 genera), and the top 1 predominant ASV was ASV_ 22320 (Supplementary Figure S2A). Statistical differential analysis revealed 422 differential ASVs between the LDH and NC groups, including 17 differential ASVs from the top 30 predominant ASVs (Supplementary Figure S2A). There were 86 differential ASVs (p < 0.05 and relative abundance >0.05%) between the two groups, and they belonged to 5 phyla, namely, *Firmicutes*, *Bacteroidota*, *Desulfobacterota*, *Actinobacteriota* and *Verrucomicrobiota*. The 30 most predominant differential ASVs belonged to 21 genera. In addition, the relative abundances of ASV_18868, ASV_15596 and ASV_14268 were significantly lower in the LDH group than in the NC cohort, whereas the relative abundances of ASV_4889, ASV_26251 and ASV_19896 were significantly higher in the LDH cohort than in the NC cohort (Figure 2E). These findings indicated alterations in the taxonomic composition of the gut microbiota in the LDH cohort.

### Taxonomic biomarkers of the gut microbiome in LDH

Based on the abundance and evolutionary relationships of the gut microbiome, linear discriminant analysis (LDA) effect size (LEfSe) and LDA methods were performed to identify gut fecal microbial biomarkers from the phylum level to the genus level between the LDH cohort and the NC cohort. The gut fecal microbial biomarkers of the NC group at different taxonomic levels included the phyla *Bacteroidota*(including *Bacteroidia*, *Bacteroidales*, *Bacteroidaceae* and *Bacteroides*) and *Oscillospirales* (including *Ruminococcaceae* and *Faecalibacterium*), whereas the biomarkers of the LDH group included the phyla *Actinobacteriota* (including *Actinomycetales*, *Actinomycetaceae* and *Actinomyces*), class *Bacilli* (including *Lactobacillales*, *Streptococcaceae* and *Streptococcus*) and *Clostridiales* (including *Clostridiaceae* and *Clostridium_sensu_stricto_1*) (Figures 3A,B). Moreover, *Parabacteroides*, *Faecalibacterium*, *Phascolarctobacterium*, *Roseburia*, *Oscillibacter*, *Butyricimonas*, *Odoribacter*, *Alistipes* and *Lachnospiraceae_UCG−010* were significantly enriched in the NC group compared to the LDH group (Figure 3C). These microbes are probiotics, and they play potential roles in anti-inflammatory effects and have protective functions in various diseases and overall human health. In contrast, *Streptococcus*, *Actinomyce*s, *Granulicatella*, *Solobacterium*, and *Clostridium_sensu_stricto_1*, *Eggerthella*, *Rothia*, *Erysipelatoclostridium*, and *[Ruminococcus]_gnavus_group* were highly enriched in the LDH group compared with the NC group (Figure 3C). These microbes have potential pathogenic or proinflammatory roles. These potential beneficial or pathogenic bacteria may be involved in the pathological mechanisms of LDH, suggesting that they may provide therapeutic strategies for LDH patients.

**FIGURE 3:**
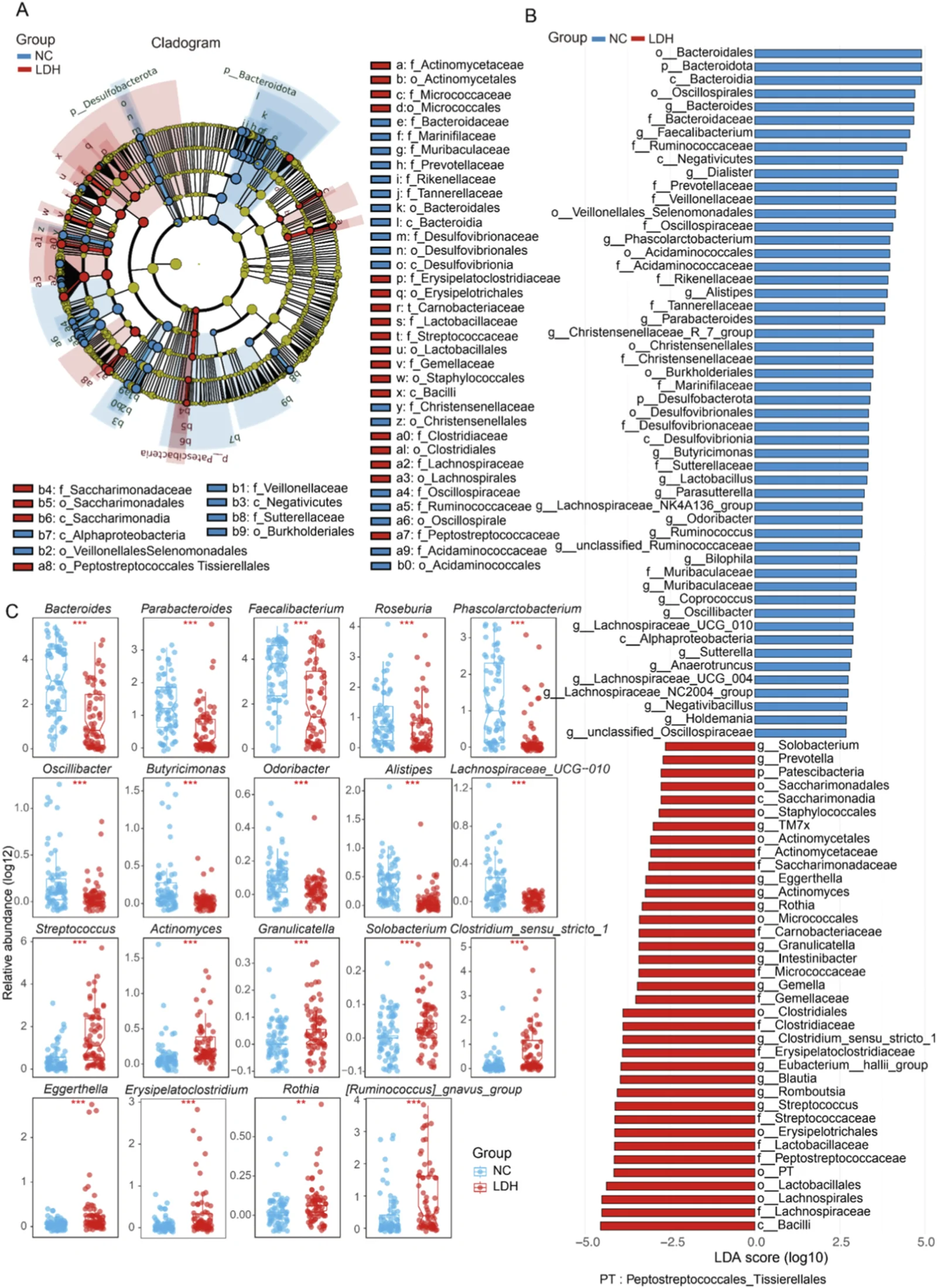
Biomarkers of gut microbes in the LDH or NC group **(A)** Cladogram based on the relative abundance of ASVs and microbial phylogenetic relationships displaying the taxonomic differences between the LDH and NC groups. The layers from inside to outside represent the phylum, class, order, family and genus levels. Red and blue bars represent taxa enriched in the LDH and NC cohorts, respectively. **(B)** LDH scores for the biomarkers at different taxonomic levels (LDH >2.0). **(C)** Relative abundance (log2-transformed) of potential probiotics or pathogenic microbes. * FDR <0.05, ** FDR <0.01, and *** FDR <0.001, Wilcoxon rank-sum test.

### The LDH and NC cohorts exhibit distinct microbial community patterns and key microbes

The gut microbiome constitutes a complicated ecosystem where certain keystone microbes play important roles in the bacterial community (Torres-Sánchez et al., 2023). Co-occurrence networks are used to assess the various co-occurrence patterns within the gut bacterial community (Tamana et al., 2021; Cui et al., 2022). Therefore, the co-occurrence networks of the NC and LDH cohorts at the genus and ASV levels were constructed in the present study. At the genus level, the gut fecal bacterial co-occurrence network of the NC group consisted of 214 edges and 90 nodes, while that of the LDH group consisted of 326 edges and 105 nodes (Figures 4A,B). To further validate the divergence of the bacterial co-occurrence network between the two groups, network-level topological features, such as the weighted degree and clustering, were evaluated. Compared with the NC bacterial co-occurrence network, the LDH bacterial co-occurrence network had higher weighted degree, clustering and betweenness centrality but lower statistical inference (Supplementary Figure S3A).

**FIGURE 4:**
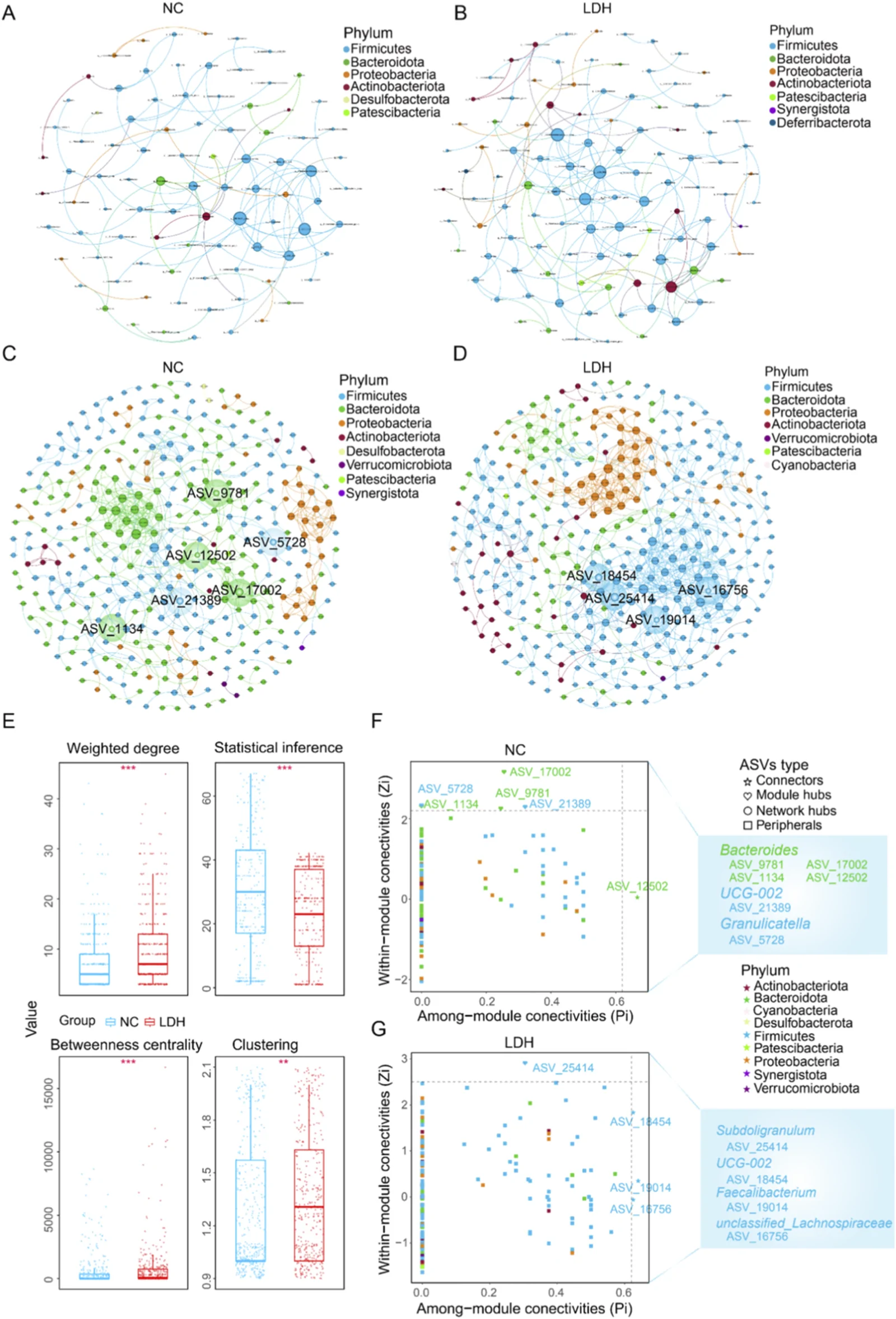
Bacterial co-occurrence networks and keystone microbes in the NC and LDH groups **(A,B)** Genus co-occurrence network between the NC **(A)** and LDH **(B)** groups based on correlation analysis. The node size indicates the linking number of a node, namely, the degree. The colors of the nodes represent the different phyla. **(C,D)** ASV co-occurrence network of the NC **(C)** and LDH **(D)** groups based on correlation analysis. The node size indicates the linking number of a node, namely, the degree. The colors of the nodes represent the different phyla. The nodes in the center represented by larger circles indicate the keystone ASVs in the co-occurrence network. The larger circles highlight the keystone ASVs, and their colors follow the node colors. **(E)** Estimates of topological features in the co-occurrence network between the two groups. **(F,G)** Classification of keystone ASVs in the co-occurrence networks of the NC **(E)** and LDH **(F)** groups. The taxonomy of keystone ASVs is provided in the rectangles. The colors of theASVs represent the different phyla. The shapes of theASVs indicate the different classifications (such as connectors, module hubs, network hubs and peripherals).*FDR <0.05, ** FDR <0.01, and *** FDR <0.001, Wilcoxon rank-sum test.

At the ASV level, the gut fecal microbial co-occurrence network of the NC group consisted of 1214 edges and 371 nodes, whereas that of the LDH group consisted of 1642 edges and 360 nodes (Figures 4C,D). Compared with those in the NC bacterial co-occurrence network, the weighted degree, clustering and betweenness centrality in the LDH network were significantly increased, whereas the statistical inference was significantly decreased in the LDH network (Figure 4E). These data indicated that the microbial community pattern of the LDH microbial co-occurrence network is distinct from that of the NC microbial co-occurrence network.

In addition to the general co-occurrence network characteristics, the topological features of individualASVs provide valuable insights into their ecological roles within the gut bacterial community (Li K. et al., 2024). In the present study, the ASVs were clustered into four major modules based on the within-module connectivity (Zi) and among-module connectivity (Pi) in the microbial co-occurrence network (Figure 4). The keystone microbes in the NC network were driven mainly by the phylum *Bacteroidota* (Figure 4F), and the keystone microbes in the LDH network were driven by the phylum *Firmicutes* (Figure 4G). In detail, the genera *Bacteroides* (4 ASVs), *Oscillospiraceae UGC-002* (1 ASV) and *Granulicatella* (1 ASV) were keystone microbes in the NC microbial network (Figure 4F), whereas *Faecalibacterium* (1 ASV), unclassified_*Lachnospiraceae* (1 ASV), *Subdoligranulum*(1ASV) and *Oscillospiraceae* UGC-002 (1ASV) were keystone microbes in the LDH microbial network (Figure 4G). Overall, the keystone biomarkers of the LDH co-occurrence network were distinct from those of the NC group, thereby providing new insights into the pathological mechanisms of LDH.

### Identification of the specific metabolic and related pathways in LDH

The PICRUSt2 tool was employed to explore functional and metabolic-related alterations in the gut fecal microbiome of LDH patients. According to the PCoA, there were significant differences between the two cohorts based on the abundance of KEGG Orthology (KO) identifiers or Enzyme Commission (EC) numbers (Figures 5A,B). The Wilcoxon rank-sum test revealed 2389 differential KO identifiers and 747 differential EC numbers between the two groups. At the first KEGG pathway level, metabolism, genetic information processing, environmental information processing and organismal systems were abnormal between the two cohorts (Supplementary Figure S4A). At the second KEGG pathway level, 28 pathways, including the metabolism of protein families, amino acids, energy, cofactors, vitamins, nucleotides, glycans and xenobiotics, as well as membrane transport, signal transduction, endocrine system and immune system, were significantly different between the two groups (Supplementary Figure S4B). At the KEGG pathway level, the metabolism of amino acids, tryptophan, phenylalanine, beta-alanine, tyrosine, ascorbate, aldarate, benzoate and sulfur, as well as protein kinases, replication, repair, transcription factors and transcription, were more enriched in the LDH group, whereas the pathways of protein phosphatases and associated proteins, lysosomes, sphingolipid metabolism, glutamatergic synapses, GABAergic synapses, other glycan degradation, PPAR signaling, replication and repair were more abundant in the NC group (Figure 5C). At the EC level, the biosynthesis of L-alanine, L-lysine, L-threonine and L-methionine, as well as sucrose degradation III and the aspartate super pathway, were significantly increased in the LDH group compared with the NC group, whereas the biosynthesis of GDP-mannose, nucleotides, flavin, polyisoprenoid and queuosine, as well as starch degradation V, gluconeogenesis I, the TCAcycle, fatty acid elongation (saturated) and mixed acid fermentation, were significantly decreased in the LDH group compared with the NC group (Supplementary Figure S4C).

**FIGURE 5:**
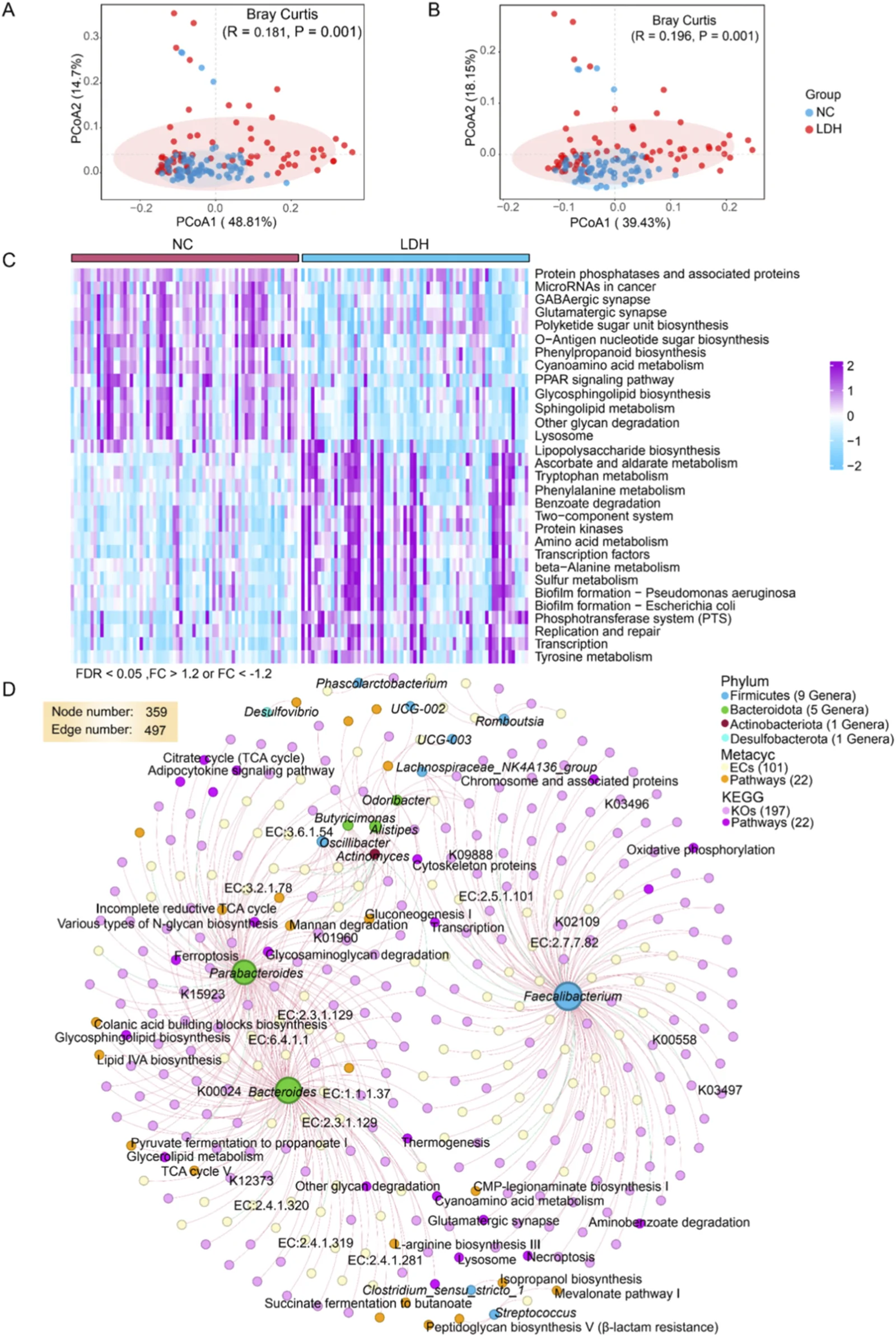
Gut microbes are correlated with functional and metabolic alterations in LDH **(A,B)** PCoAof KO identifiers **(A)** and EC numbers **(B)** based on the Bray‒Curtis dissimilarities for the LDH and NC groups (ANOSIM test). **(C)** Heatmap of the 30 most differential KEGG level 3 pathways based on the relative abundance of pathways between the two groups. **(D)** The correlation network displays strong correlations between the dominant differential microbiota and metabolic pathways, EC numbers or KO identifiers. The node size indicates the degree (topological feature) of the network. The colors of the nodes represent the different phyla, KO identifiers, EC numbers and predicted metabolic pathways. The red edges indicate positive interactions, and the green edges represent negative interactions between nodes. * FDR <0.05, ** FDR <0.01, and *** FDR <0.001, Wilcoxon rank-sum test.

To investigate the correlation between the gut microbiota and ECs, KOs or predicted pathways, the gut fecal bacterial correlation network was constructed. The correlation network consisted of 16 dominant genera, such as *Faecalibacterium*, *Bacteroides*, *Parabacteroide*s, *Alistipes*, *Romboutsia* and *Streptococcus*, from the top 30 genera (Figure 2C), which belong to the phyla *Firmicutes* and *Bacteroidota*. Moreover, 101 ECs and 22 metabolic pathways were predicted with the Metacyc database, while 197 KOs and 22KEGG pathways were predicted with the KEGG database. Further, the correlation network was comprised of 359 nodes and 497 edges (Figure 5D). The present data revealed that the genus *Bacteroides* was highly correlated with 22 pathways (including the TCAcycle, mannan degradation, and gluconeogenesis I), 40 ECs (including EC:1.1.1.37 and EC:2.4.1.281) and 75 KOs (including K12373 and K15923) in the network. The EC database showed that 4 enzymes (EC:2.4.1.281, EC:2.4.1.319, EC:2.4.1.320 and EC:3.2.1.78) were involved in the mannan degradation pathway and that these 4 enzymes were also positively correlated with *Bacteroides* (Figure 5D), suggesting that *Bacteroides* may regulate the mannan degradation pathway via these 4 enzymes. Similarly, the KEGG database revealed that the K01960 and K00024 enzymes participated in the citrate cycle (TCAcycle). Furthermore, the genera *Parabacteroides* were positively correlated with K01960, K00024 and the citrate cycle, indicating that *Parabacteroides* may regulate the citrate cycle via the K01960 and K00024 enzymes (Figure 5D). Similarly, *Faecalibacterium*, which was one of the dominant genera in the correlation network, was positively correlated with the cytoskeleton protein pathway. In addition, the K03497 enzyme was also involved in the cytoskeleton protein pathway according to the KEGG database, suggesting that *Faecalibacterium* may positively regulate the cytoskeleton protein pathway by K03497 (Figure 5D). Although the predominant gut microbiota may regulate its associated metabolic pathways through related KO identifiers and EC numbers, further experiments are needed for additional exploration and verification of these findings.

### A new model for the clinical diagnosis of LDH with metabolic and related pathways

To explore whether the gut fecal microbes, ADIs, KO signals and EC enzymes of the LDH cohort differentiate from those of the NC cohort, random forest (RF) classifiers were performed on the basis of previously identified genus microbes, alpha diversity indices (ADIs), KO identifiers and EC numbers. Tenfold cross-validation of the RF models was performed, and receiver operating characteristic (ROC) curves were generated to classify LDH patients and NC subjects. The RF model with 14 predictors had the optimal performance, and *Butyricimona*s was the top predictor in this model (Figures 6A,B); the area under the curve (ROC) value was 0.946 (Figure 6C). In addition, the RF model with all 7 ADIs achieved good performance, and ACE was the predominant predictor in this model (Supplementary Figures S5A,B); the ROC value was 0.843 (Supplementary Figure S5C). Moreover, the optimal RF model using 21 KO signals or 30 EC numbers achieved good performance, and K16363 (*LpxC-fabZ*) or EC:2.5.1.46 (deoxyhypusine synthase) was the predominant predictor in this model (Supplementary Figures S5D,E,G,H); the ROC values with K16363 and EC:2.5.1.46 were to 0.942 and 0.956, respectively (Supplementary Figures S5F,I). Overall, these results demonstrated that the RF model using single-type features, including metabolic and related pathways, is feasible and effective in discriminating potential LDH patients from healthy individuals.

**FIGURE 6:**
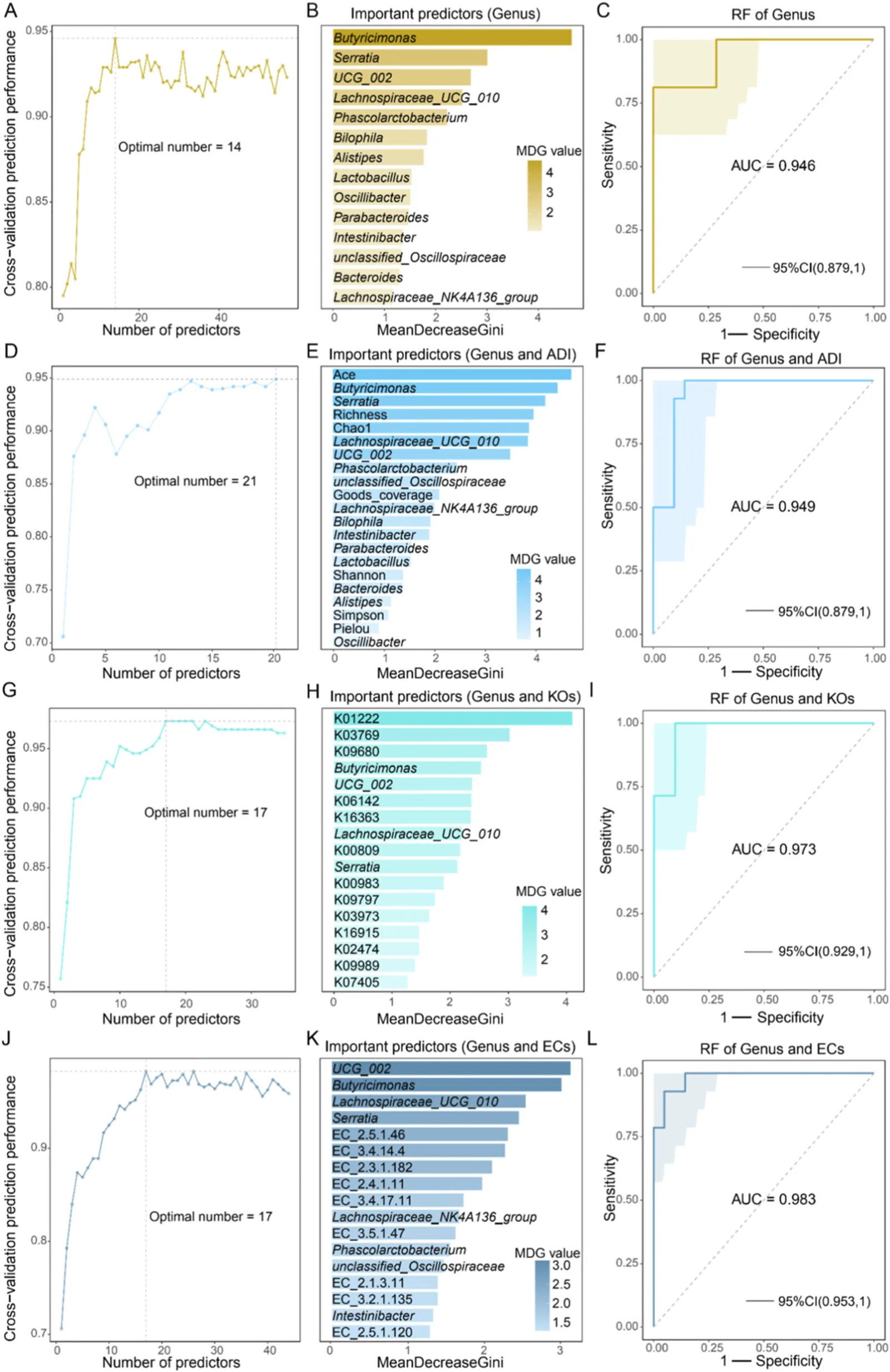
Discriminative performance of the random forest model based on genera, alpha diversity indices (ADIs), KO identifiers, and EC numbers. **(A,D,G,J)** Predicted performance of the RF model using optimal feature sets: genera alone **(A)**, 14 genera combined with 7 ADIs **(D)**, genera combined with KO identifiers **(G)**, and genera combined with EC numbers **(J)**. **(B,E,H,K)** Feature importance rankings (by MeanDecreaseGini values) for the optimal feature sets: 14 genera **(B)**, 14 genera +7 ADIs **(E)**, 4 genera +13 KO identifiers **(H)**, and 8 genera +9 EC numbers **(K)**. **(C,F,I,L)** Receiver operating characteristic (ROC) curves for the RF model based on the corresponding optimal feature sets. AUC: area under the curve; ADIs: alpha diversity indices; KO: KEGG Orthology; EC: Enzyme Commission. Statistical significance was assessed using the Wilcoxon rank-sum test with FDR correction: *FDR <0.05, **FDR <0.01, ***FDR <0.001.

When gut microbial features were combined with alpha diversity indices (ADIs), the RF model incorporating 21 predictors (7 ADIs and 14 genera) showed a modest improvement in performance compared with the model based on genera alone (Figures 6D,E). The ACE index emerged as the most influential contributor in this combined model (Figure 6E), which achieved an ROC value of 0.949 (Figure 6F). Furthermore, the RF model with 17 variables (4 genera and 13 KO identifiers) yielded an ROC value of 0.973, while another 17-variable model (8 genera and 9 EC numbers) achieved an ROC value of 0.983, both outperforming the genera-only model (Figures 6G–L). Collectively, these results indicate that integrating gut microbiome signatures with complementary data types—such as KO identifiers and EC numbers—substantially enhances the accuracy and effectiveness of the RF model in distinguishing LDH patients from healthy controls. This integrative approach may offer a novel strategy for LDH diagnosis and potentially facilitate early intervention in clinical practice.

## Discussion

By analyzing the gut microbiota of Chinese LDH patients, the present study demonstrated that the low abundances of gut *Faecalibacterium* and *Bacteroides* are correlated with LDH. The present data suggested that mannan degradation and cytoskeleton assembly regulated by *Faecalibacterium* and *Bacteroides* may constitute a new mechanism for LDH, and some new metabolites, including phenylalanine and β-alanine, may be involved in LDH regulation. Moreover, the random forest (RF) model for clinical LDH diagnosis combined with those specific pathways and metabolites presented good accuracy. These results provide deeper mechanistic insight into LDH progression and potential drug targets for the clinical diagnosis and treatment of Chinese LDH patients.

Multiple factors have been implicated in the initiation and progression of LDH, including obesity (Lurie et al., 2014; Qu et al., 2023), inflammation (Albert et al., 2024), and anxiety (Moon et al., 2023). The gut microbiota may act as a central mediator linking these factors to LDH pathogenesis. Recent studies have shown that patients with type 2 diabetes mellitus exhibit reduced levels of Bacteroidetes (Martínez-Carrillo et al., 2024), suggesting that type 2 diabetes may influence LDH through mechanisms dependent on Bacteroidetes abundance. Similarly, the relative abundance of Faecalibacterium is significantly lower in smokers compared with nonsmokers (Yan et al., 2021), implying that smoking may contribute to LDH risk. Furthermore, a significant decrease in Bacteroidetes abundance has been observed in Chinese patients with sleep-disordered breathing (Yang et al., 2024), highlighting the potential involvement of central nervous system disturbances in LDH. Collectively, these findings suggest that previously underappreciated factors—such as glycemic status and smoking—may represent novel pathways through which gut microbiota alterations influence LDH progression, potentially mediated by changes in the abundance of Faecalibacterium and *Bacteroides*.

In addition to the low abundances of *Faecalibacterium* and *Bacteroides* observed in the LDH cohort, *Streptococcus*, *Actinomyces*, *Eggerthella*, *Ruminococcus gnavus* group and *Clostridium*_ sensu_ *stricto_1* were increased in LDH patients, which may trigger autoimmune diseases and drive senescence (Walker et al., 2014; Brooks and Mias, 2018). These phenomena cannot be ignored when investigating alterations in the gut microbiota and LDH progression. The present results indicated that the microbial community pattern of the LDH microbial co-occurrence network was distinct from that of the NC microbial co-occurrence network. These keystone microbes, including *Faecalibacterium* and *Bacteroides*, may also provide new insights into the pathological mechanisms of LDH.

The present data verified that many neuropathological activities, including GABAergic and glutamatergic synapse activation, were significantly altered in LDH patients, which is consistent with the previously reported key role of LDH initiation (Aoki et al., 2006). The present study also demonstrated that other intracellular signals, such as mannan degradation and cytoskeleton assembly, may affect LDH progression. Recently, mannose metabolism has been reported to reshape T cell differentiation to enhance antitumor immunity (Qiu et al., 2024), and the polarization of T lymphocytes is involved in the pathogenesis of LDH (Yao et al., 2017), indicating that mannan degradation regulation may be the mechanism for LDH regulation by T cells. Cytoskeleton assembly is a dynamic network consisting of actin filaments, microtubules (MTs), and intermediate filaments (IFs) (Li et al., 2023). Changes in cytoskeleton assembly are associated with many diseases, including nonsyndromic congenital cataracts and DOCK8 deficiency (Papa et al., 2020), but the correlation between cytoskeleton assembly and LDH is unknown. The present data also provide evidence that alterationsin the gut microbiota may affect nucleuspulposuscy to skeleton assembly, further regulating LDH initiation and progression. These new pathways lay a foundation for a better understanding of the mechanism of LDH and the discovery of new drug targets for LDH treatment.

Several limitations of this study should be acknowledged. First, the relatively small sample size may limit the statistical power and generalizability of the sequencing results. Second, the absence of *in vivo* and *in vitro* experimental validation in LDH animal models restricts our ability to draw definitive conclusions regarding the functional roles of the gut microbiota and its metabolites in disease pathogenesis. Third, as all participants were recruited from a single geographic region in China, the findings may not be directly generalizable to other populations due to differences in dietary habits, environmental exposures, and geographical factors.

In summary, this study provides evidence that gut microbial dysbiosis—characterized by reduced abundances of Faecalibacterium and Bacteroides—is associated with LDH in Chinese patients. We also identified several novel metabolites and signaling pathways that may be involved in LDH regulation, offering candidate biomarkers and potential therapeutic targets for future investigation. However, further functional studies are warranted to validate these findings and to elucidate the underlying mechanisms.

## Conclusion

The gut microbiota is increasingly recognized as a critical modulator of diverse physiological and pathological processes; however, its compositional and functional profile in patients with lumbar disc herniation (LDH)—particularly in the Chinese population—has remained largely unexplored. In this study, we performed 16S rRNA sequencing on a cohort of 69 LDH patients and 69 matched healthy controls, and integrated untargeted metabolomic profiling to comprehensively characterize the gut microbial and metabolic alterations associated with LDH. Our results revealed significant differences in both alpha and beta diversity between the two groups, indicating a profound shift in gut microbial ecology at the onset of LDH. Taxonomic, phylogenetic, and co-occurrence network analyses further demonstrated that reduced abundances of Faecalibacterium and *Bacteroides* were strongly correlated with LDH. Beyond the classical proinflammatory response and suppression of glutamatergic and GABAergic neuronal signaling, our integrative analysis uncovered that these two depleted genera may contribute to LDH pathophysiology through previously unrecognized mechanisms, specifically involving the regulation of mannan degradation and cytoskeleton assembly. In addition, we identified phenylalanine and β-alanine as novel metabolites potentially involved in LDH regulation. Leveraging these pathway and metabolite signatures, we developed a random forest-based diagnostic model that achieved good predictive accuracy, highlighting its potential as a non-invasive clinical screening tool. Collectively, our findings provide the first evidence in a Chinese population linking gut microbiota dysbiosis to LDH, offer new mechanistic insights and metabolic mediators, and present a refined strategy for improving LDH diagnosis.

## Data Availability

The datasets presented in this study can be found in online repositories. The names of the repository/repositories and accession number(s) can be found in the article/Supplementary Material.
