# Supplementary material for: Identifying the key microbiome associated with lumbar discherniation in Chinese patients
Source: Front Cell Dev Biol. 2026 Sep 15;14:1942642. doi: 10.3389/fcell.2026.1942642 (PMC13619939; doi:10.3389/fcell.2026.1942642)
Supplement: Supplementary file 1 [file DataSheet1.docx]

**Figure S1. The shift of gut microbiome between two groups**

(A, B) The estimates of Chao1 index (A) and Richness index (B). (C) There was no significant difference in ratio of F/P between two groups. (D) There was no significant difference in relative abundance of *Proteobacteria* between two groups.

**Figure S2. Sankey diagram of top 30 predominant ASVs based on relative abundance of ASVs between groups.** The first column represents group, namely red for LDH and Blue for NC. The second column represents top 30 predominant ASVs. The third and fourth column stands for genus and phylum, respectively.

**Figure S3. Estimates of topological features in the co-occurrence network based on genera between two groups.**

**Figure S4. The predicted** **differential** **metabolic pathways of KEGG Level 1, KEGG Level 2 and EC.**

(A-B) The differential KEGG Level 1 (A) and Level 2 (B) metabolic pathways based on the relative abundance of pathways between two groups. (C) The heatmap of 30 most important differential EC pathways based on the relative abundance of pathways between two groups.

**Figure S5. Remarkably discriminating LDH and NC cohorts by ADIs, KOs and ECs.**

(A, D, G) Predicted performance of a RF model with optimal and important features, such as ADIs (A), KOs (D), ECs (G). (B, E, H) The optimal features, namely 7 ADIs (B), 21 KOs (E), 30 ECs (H), in RF model ordered by MeanDecreaseGini values. (C, F, I) ROC curves for the RF model based on the optimal features. AUC, area under curve. ADIs, alpha diversity indexes.
